# Systematic Review and Meta-Analysis on Knowledge Attitude and Practices on African Animal Trypanocide Resistance

**DOI:** 10.3390/tropicalmed7090205

**Published:** 2022-08-23

**Authors:** Keneth Iceland Kasozi, Ewan Thomas MacLeod, Charles Waiswa, Michael Mahero, Ibrahim Ntulume, Susan Christina Welburn

**Affiliations:** 1 Infection Medicine, Deanery of Biomedical Sciences, College of Medicine and Veterinary Medicine, The University of Edinburgh, Edinburgh EH8 9JZ, UK; 2 School of Medicine, Kabale University, Kabale P.O. Box 317, Uganda; 3 School of Biosecurity Biotechnical and Laboratory Sciences, College of Veterinary Medicine Animal Resources and Biosecurity, Makerere University, Kampala P.O. Box 7062, Uganda; 4 Department of Veterinary Population Medicine, College of Veterinary Medicine, University of Minnesota, Minneapolis, MN 55108, USA; 5 Zhejiang University-University of Edinburgh Institute, Zhejiang University School of Medicine, International Campus, 718 East Haizhou Road, Haining 314400, China

**Keywords:** trypanocide resistance, trypanosomiasis, human African trypanosomiasis, animal African trypanosomiasis, one health, global health, community practices attitudes and practices, AATr

## Abstract

Background: African trypanocide resistance is an emerging public health emergency whose control requires a revisit on farmer’s knowledge, attitudes, and practices in developing countries. African animal trypanocide resistance (AATr) is rife in an environment where drug use and policy decisions are disjointed. The objective of the study was to identify community factors responsible for the development of AATr. This was important since diminazene aceturate (DA), isometamidium chloride (ISM), and homidium bromide (HB) have existed for over 30 years and no new drugs have been provided to farmers. Methods: An electronic keyword search across 12 databases was conducted using a search criterion from 1806 to June 2022. This generated a total of 24 publications, but after removing duplicates, review articles, and nonrelated articles, a total of eight papers were included in the analysis by following the PRISMA checklist. A meta-analysis was conducted on the data extracted and the risk ratio and inverse variance at 95% confidence interval were calculated using RevMan^®^. Results: All the eight articles in the study showed that DA was the most preferred trypanocide in both West and Eastern Africa. Poor farmer knowledge of AATr and limited drug options were major drivers for trypanocide resistance. In addition, farmer treatments, use of untrained personnel, poor administration, poor dosing, and preparation of trypanocides were major drivers for the development of AATr and similarities were identified in DA and ISM practices (P = 0.13). Conclusions: AATr is spread in developing countries due to a lack of community knowledge, attitudes, and drug-use practices. This situation could be reversed through interdisciplinary collaborations in endemic communities by promoting effective treatments and responsible drug handling.

## 1. Introduction

In sub-Saharan Africa, there have been three leading trypanocide compounds from 1950 to date (i.e., homidium bromide (HB) in 1952, diminazene aceturate (DA) in 1955, and isometamidium chloride (ISM) [1]). For the control of African trypanosomiasis, the international community needs to prioritise and generate funding for the production of new trypanocide compounds [2]. A decision in this direction could help reverse the currently high African animal trypanocide resistance (AATr) in several developing countries [3]. Critical weaknesses in human resources and technology have led to the continuously poor surveillance of neglected zoonotic diseases (NZD), demonstrating the importance of policymakers in capital distribution for the development of medical research in endemic communities. The lack of initiative has created an atmosphere where NZD continues to proliferate with new genetic mutations taking place (see [4] on trypanosomes), drug abuse, and the development of drug resistance due to weak pharmacovigilance policies. 

In Africa, farmers have continued to handle trypanocides with or without any supervision from veterinarians. Drug abuse remains a problem on most farms because they lack appropriate theoretical knowledge on the mechanism of action of the drugs, administration, and dosage, and are frequently unable to read the pharmaceutical information on the leaflets. This situation arises when farmers rely heavily on visual cues and body condition score to make a diagnosis and from past experiences with related diseases while administering medications. For example, in Tanzania, DA is always abused as it is prophylactically administered for control of tick-borne diseases (TBD) in the community [5]. Similar findings have been found in Uganda, where the low prevalence of trypanosomes in some communities has been associated with the frequent use of pyrethroids and restricted grazing with or without trypanosome disease burden [6]. 

African trypanosomiasis control in Africa has also been hampered by the low or poor community attitudes towards trypanocide usage. In Tanzania, adoption of novel disease control strategies by farmers failed since they perceived these as being costly [7]. This atmosphere is precipitated by the high illiteracy levels and poor extension services on NZD in rural African communities. For example, Ugandan communities (Arua) expressed fear when they saw tsetse baits for the first time, confusing them for “witchcraft” and “ghosts from the river” which are conventionally allied with mental illness, death, and misfortune [8].

African trypanosomiasis endemic status in most African countries has moved farmers to resort to general vector control methods that appear simple, cheap, and suitable for riverine tsetse species [9]. This is because the approaches employed by farmers are often not recommended by the local government veterinarians (extension workers), such as extensive dipping, bush burning, fires, especially in the dry seasons, traps-impregnated, and chemical sprays using acaricides [7]. For example, farmers in Cameroon applied insecticides 2–3 times a week [10], while in Uganda (see [6] in Mbarara), and Tanzania (see [5] in the east and northeastern regions), farmers used pyrethroids and trypanocides without veterinary prescriptions. These practices continue to create an atmosphere where AATr will continue to develop unless there is a change in community knowledge and practices promoting responsible drug usage.

In this study, we provide information highlighting critical drivers responsible for the development of African animal trypanocide resistance in developing countries. In particular, emphasis was placed on community knowledge on drug usage, attitudes and practices on drug misuse, drug failure, and development of resistance. This was important to help revive international momentum for the control of trypanosomiasis and motivate African countries to improve legislation on drug protection.

## 2. Methods

An electronic keyword search using the phrases; All = ((African trypanosomiasis or bovine trypanosomiasis or animal African trypanosome asis or trypanosoma brucei or trypanosoma adja brucei brucei or trypanosoma evansi or trypanosoma vivax or trypanosoma congolense)) AND ALL = (knowledge attitude and practices) AND ALL = (drug resistance or trypanocides resistance or resistant trypanosomes or hormidium achloriden or hormidium bromide or melarsamine or quinpramine or isometamidium chloride), were applied across 12 databases (eleven from Ovid and one from Web of Science), i.e., Database Field Guide AMED (Allied and Complementary Medicine), from 1985 to May 2022, Database Field Guide CAB Abstracts from 1973 to week 21, 2022, Database Field Guide APA PsycInfo from 18 June to May week 4, 2022, Database Field Guide Books@Ovid 31 May 2022, Database Field Guide Journals@Ovid full text, 31 May 2022, Database Field Guide Your Journals@Ovid, Database Field Guide APA PsycArticles full text, Database Field Guide CAB Abstracts from 1910 to 1989, Database Field Guide Embase Classic + Embase from 1947 to 31 May 2022, Database Field Guide Global Health from 1910 to week 21, 2022, Database Field Guide Ovid MEDLINE(R) and Epub Ahead of Print, in-process, in-data-review and other non-indexed citations, daily and versions from 1946 to 31 May 2022, (n = 16), and Web of Science (n = 1) on 1 June 2022 (see Supplementary Materials).

### 2.1. Inclusion and Exclusion Criteria on Research Articles

**Figure 1:**
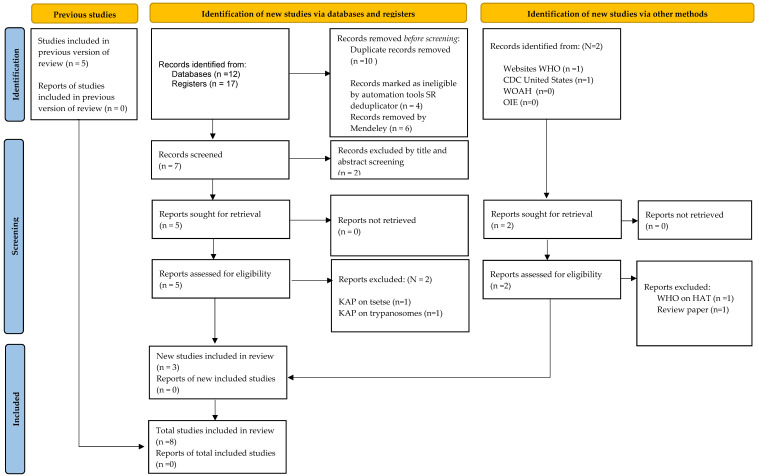
PRISMA checklist showing articles identified from the databases, Google, and in-citation search on knowledge, attitude, and practices on African animal trypanocide resistance.

Data was exported to Mendeley, and all papers were merged (n = 17), removing a total of 6 duplicates. The SR depublicator removed another 4 duplicates, leaving 7 papers. These were then exported to Covidence for article screening by all authors (Figure 1). Further studies were searched from previous studies by looking at the references, and we identified 5 studies, while 2 reports were added (and latter removed) to the collection from a Google search from the World Health Organization (WHO), World Organization for Animal Health (WOAH), and from the Organization for Animal Health (OIE), US Centers for Disease Control (CDC). After excluding studies on AAT knowledge, attitude, and practices, a total of eight studies were included in the data analysis.

### 2.2. Statistical Analysis

Data extracted from studies was entered into MS Excel and subsequently imported into RevMan^®^ for meta-analysis. The inverse variance was calculated, while the risk ratio was used to measure the effect size at a 95% confidence interval. The analysis model used the random effects model since these studies were conducted in different countries under varying conditions

## 3. Results

### 3.1. Description of Papers in the Study on Community Knowledge Attitude and Practices in African Animal Trypanocide Resistance

A total of eight articles (n = 8) on African animal trypanocide resistance (AATr) knowledge, attitudes, and practice community surveys were included in the study. Most of the studies have been done in West Africa (i.e., Cameroon, Burkina Faso, Mali, and Guinea) and East Africa (Ethiopia and Kenya). There was a very high adoption of diminazene aceturate (DA) as compared to isometamidium chloride (ISM), and practices in rural communities are critical for the propagation of AATr (Table 1). 

### 3.2. Community Knowledge and Attitudes on African Animal Trypanocide Resistance

In Tanzania, the community had excellent knowledge of tsetse [5] and in Kenya, farmers (90%, 585/650) were aware of trypanosomiasis and appropriate measures for disease control [12], although this did not reflect their knowledge of trypanocide resistance. Farmers have cited reinfection, immune compromised status of the animal, drug resistance, and poor quality drugs as sources of drug failure in West Africa, i.e., in Burkina Faso, Mali, and Guinea [13].

In Tanzania, farmers reported unavailability of drug options, unwillingness to control the disease in the community by other farmers, poor knowledge and lack of veterinary services, as well as financial constraints, as major drivers for the abuse of trypanocides [5]. In West Africa, tsetse were recognized as important causes of AAT (83.9%, 751), poor immune status due to malnutrition (60.3%, 540/896), and sick animals were identified as major risk factors in the herd (27.7%, 248/896), justifying the use of pyrethroids on farms [13]. Furthermore, DA was preferred over ISM since it was cheaper, easy to use, and accessible in Ethiopia [15].

### 3.3. Meta-Analysis on Community Practices and Control Measures against African Animal Trypanocide Resistance

In Mali, promoting trypanotolerant breeds is a common practice to address drug resistance [16]. In Cameroon, 90% (n = 64) treated the animals themselves and only 5% relied on veterinary service providers. These were identified as being responsible for the high DA and ISM resistance in the community [10]. Similar reports have been reported in Kenya, where 73% (475/650) of treatments were done by untrained personnel [12]. A high degree of variation in trypanocide usage and the intervals of their application (i.e., 20% of respondents rely on trypanocide for trypanosomiasis control [5] in Tanzania) have been reported, demonstrating an absence of standard treatment regiments. In addition, the majority (95%) wrongly used DA, thinking it was prophylactic, while only 30% used ISM correctly. Most of the respondents administered the drugs on their own, while in some cases, these medications were not administered properly (did not follow the manufacturer’s manual), i.e., intravenous routes and no observance of withdrawal periods. In Ethiopia, the community preferred mainly DA vs. ISM for treatment of AAT (79 vs. 21%), practised overdose, relied on unqualified people to administer treatments and sourced drugs from unauthorized outlets [15]. These findings were reproduced in a recent study in the Omo Zone of South Western Ethiopia [14]. In the same study area (Omo Zone, Ethiopia), DA was mainly used rather than ISM, and most of the animals were overdosed [11]. 

**Figure 2:**
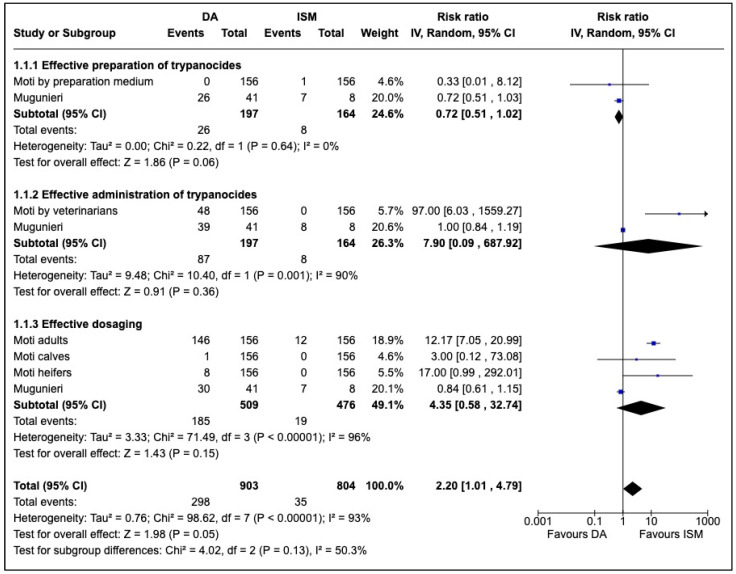
Meta-analysis of community practices responsible for the development of African trypanocide resistance.

Meta-analysis revealed that effective preparation and administration of trypanocides were reported in favour of DA by veterinarians (Figure 2). DA was administered more than eight times correctly compared to ISM while dosing was four times better with DA rather than with ISM, although practices on DA and ISM were significantly identical (RR = 2.20, 95% CI: 1.01–4.79).

## 4. Discussion

In this study, we provide evidence that the development of AATr is related to community knowledge, attitudes, and practices. Most of these studies have been conducted in West and Eastern Africa since these are endemic regions along the sub-Saharan belt. Community knowledge was found to be weak since this did not translate to practice. In Tanzania, farmers reported unavailability of drugs, lack of initiative to control the disease in the community, poor knowledge, and lack of veterinary services as well as financial constraints as major drivers for the abuse of trypanocides [5] while in Ethiopia, DA was preferred over ISM since it was cheaper, easy to use and access [15]. In western Kenya, improved farmers’ knowledge (see [17] following over 40 years of interactions with the same compounds) on trypanocides was associated with a poor prognosis in farmer-led-animal treatments, leading to the development of trypanocide resistance in the herds [12].

In an effort to contain AAT, local breeds which are trypanotolerant have been promoted in West Africa [16], although this does not translate to an eradication of the trypanocide resistant genotype in the community. Continued farmer treatments, substandard drugs on the market, and poor pharmacovigilance practices are critical weaknesses responsible for the proliferative AATr status in most developing countries. In Nigeria, government regulators have taken an interest in addressing trypanocide abuse in farming communities [18]. There are policy shortfalls and a lack of cohesion and harmony in regional governments which impede the management of trypanosomiasis in animals [13]. Furthermore, policy studies have shown a poor understanding of the challenges of drug resistance in most developing countries and a lack of an enabling environment for the promotion of rational drug use [16]. Establishment of political and financial commitment at the national level is essential to support national structures in developing countries to promote a progressive control pathway against trypanosomiasis [19].

The attitude of cattle keepers in a particular community has a great impact on the acceptance and use of measures required to control any epidemic. For example, most farmers in Tanzania had a positive attitude towards preventive methods. However, this was not sufficient to influence the adoption of novel disease control measures to prevent drug resistance. This was due to the perceived high costs associated with the control strategies, thus discouraging farmers from adopting them [7]. In addition, attitude is affected by knowledge; thus, low/poor knowledge of the control strategies are responsible for the weak adoption practices as farmers keep coming up with one excuse after the other. For example, communities in Northern Uganda (Arua district) lacked knowledge of community-based tsetse baits, as this conveyed panic, anxiety, and fear, because they had never seen these traps [8]. Findings in the study highlight the importance of farmer education as this offers a platform for a change in attitudes and practices. Unfortunately, this might remain challenging amidst the critical human resource shortages in the livestock industry in most developing countries. Novel strategies would have to be adopted to fill in the gaps, i.e., the adoption of the One Health paradigm or a related model where multidisciplinary and inter-institutional collaborations are promoted.

Finally, the study provides insights on the community practices that are responsible for the development of trypanocide resistance. In the study, African communities generally prefer DA over ISM, and this contributes to its overuse and abuse. During resistance, farmers continuously apply DA and ISM hoping for a miracle [20], thus worsening the situation instead of seeking professional assistance at an early stage. The continuous application of trypanocides (with or without insecticides) has created a vicious cycle of drug misuse, overuse, and subsequent drug resistance in affected communities [7,21]. The overuse of DA in healthy animals not suffering from trypanosomiasis is due to the high vitamin B12 content, which improves the animals’ blood circulation [12,22], although this does not justify its abuse since there are vitamin supplements on the market. 

The study also provides evidence that efficacy of medications was associated with reliance on veterinarians who helped with the preparation and administration of trypanocides, while the opposite (i.e., the use of untrained personnel was true for both DA and ISM) was responsible for the emerging AATr in affected communities. Under/overdosing was another mechanism through which resistance was propagated since a majority of farmers were often not keen on administering the correct dosage (often relying on carcass weight) [23] with little regard to drug routes for administration, thus causing the animals a lot of pain [5]. Similar challenges have been identified among Kenyan farmers [21] and animal health diploma graduates in Nigeria [24], demonstrating critical weaknesses in the livestock sector in most African countries. For effective control of AATr, more animals have to be treated by veterinarians using either mono or combination therapy [25].

Liberalisation of the drug industry in African countries has also been associated with the flooding of the market with fake drugs, which are often so quick to induce trypanocide resistance. Unfortunately, most of these trypanocides are provided by the private sector [15] in comparison to the government offices, which are into administrative support—leaving the farmer at the mercy of businessmen who prioritise profits above animal welfare [14]. It is important to enact strong regulatory measures in the livestock sector for the control and prevention of the spread of trypanocide resistance. The current policies for the liberalisation of the livestock section have probably been misinterpreted by most African governments, demonstrating a need to create an enabling environment to promote responsible drug use [16]. The creation of technical teams to provide evidence-based planning of field activities could help promote the elimination of AATr through the establishment of national databases to guide policy and decision making [19].

## 5. Conclusions

The development of African trypanocide resistance is multisectoral and to reverse the current trend of drug resistance, political will from policymakers is essential. It remains important to enact strong policies to ensure that rational drug use is promoted in rural communities and the practice of farmers administering treatments without prescriptions from veterinarians is stopped wherever this is applicable, especially for the control of infectious diseases. Since trypanosomiasis is a zoonotic disease, the accumulation of trypanocide resistant pathogens is a public health risk since the same genetic markers are present in animals and human infective *Trypanosoma* species. Furthermore, national governments must establish local, regional, and national databases to routinely monitor trypanocide usage and screen livestock for the development of the resistant phenotype in their local herds. This initiative could be supplemented by regional collaborative initiatives through the Common Market for West African States (COMESA), the East African Community (EAC), or the African Union. This could help create robust multidisciplinary teams which could help transform policy and community practices for the control of drug resistance for the current and future epidemics on the continent through the One Health approach.

## Figures and Tables

**Table 1:** Community surveys identified on African animal trypanocide resistance knowledge, attitudes, and practices, indicating the study area and resistance drivers.

Study	Reference	Study Area/Country	Description on AATr Status
Moti 2015	[11]	Southwestern Ethiopia	AATr in DA and ISM is associated with farmer treatments and other livestock never being treated on the farms.
Mugunieri 2003	[12]	Kwale district, Kenya	AATr in DA and ISM. Farmers’ treatments and drug abuse are responsible for resistance
Grace 2005	[13]	Burkina Faso, Mali, and Guinea	AATr in DA and ISM. Farmers had low knowledge of the disease which contributed to poor farming practices.
Tesfaye 2020	[14]	South Omo region, Southwestern Ethiopia	Farmers have a high preference for DA against ISM. Trypanocides from private drug stores are less effective than government drugs.
Tekle 2018	[15]	Southwestern Ethiopia	Farmers preferred using DA as compared to ISM. Farmers conducted treatments. Drugs sourced from illegal distributors.
Ngumbi 2017	[5]	Eastern and Northeastern Tanzania	Improper use of DA. Intravenous routes of administration and no withdrawal periods were observed.
Clausen 2010	[16]	Burkina Faso, Mali, and Guinea	Trypanotolerant cattle are kept to control AATr. Promotional drug usage through training to improve farmer knowledge and practices.
Mamoudou 2007	[10]	Cameroon	Majority of farmers treated their own animals in AATr.

Key: AATr = African animal trypanocide resistance. DA = diminazene aceturate. ISM = isometamidium chloride.

## Data Availability

All data used in the study is contained in the manuscript.

## Supplementary Materials

The Supplementary Materials are available online at https://www.mdpi.com/article/10.3390/tropicalmed7090205/s1.
