# Supplementary material for: Systematic Review and Meta-Analysis on Knowledge Attitude and Practices on African Animal Trypanocide Resistance
Source: Trop Med Infect Dis. 2022 Aug 23;7(9):205. doi: 10.3390/tropicalmed7090205 (PMC9503918; doi:10.3390/tropicalmed7090205)
Supplement: Supplementary file 1 [file tropicalmed-07-00205-s001.zip › tropicalmed-1866531-supplementary.pdf]

Tropical Medicine and Infectious Disease

MDPI

Supplementary Materials for Systematic Review and Meta-Analysis on Knowledge Attitude and Practices on African Animal Trypanocide Resistance

Supplementary Material on KAP Tryps

AMED (Allied and Complementary Medicine) <1985 to May 2022>

CAB Abstracts <1973 to 2022 Week 21>

APA PsycInfo <1806 to May Week 4 2022>

Books@Ovid <May 31, 2022>

Journals@Ovid Full Text <May 31, 2022>

Your Journals@Ovid

APA PsycArticles Full Text

CAB Abstracts <1910 to 1989>

Embase Classic+Embase <1947 to 2022 May 31>

Global Health <1910 to 2022 Week 21>

Ovid MEDLINE(R) and Epub Ahead of Print, In-Process, In-Data-Review & Other Non-Indexed Citations, Daily and Versions <1946 to May 31, 2022>

1 bovine trypanosomiasis.mp. [mp=ab, hw, ti, ot, bt, id, cc, tc, tm, mf, tx, ct, tn, dm, dv, kf, fx, dq, nm, ox, px, rx, ui, sy] 1016

2 african animal trypanosomiasis.mp. [mp=ab, hw, ti, ot, bt, id, cc, tc, tm, mf, tx, ct, tn, dm, dv, kf, fx, dq, nm, ox, px, rx, ui, sy] 397

3 animal african trypanosomiasis.mp. [mp=ab, hw, ti, ot, bt, id, cc, tc, tm, mf, tx, ct, tn, dm, dv, kf, fx, dq, nm, ox, px, rx, ui, sy] 401

4 trypanosoma brucei.mp. [mp=ab, hw, ti, ot, bt, id, cc, tc, tm, mf, tx, ct, tn, dm, dv, kf, fx, dq, nm, ox, px, rx, ui, sy] 50597

5 (trypanosoma adj vivax).mp. [mp=ab, hw, ti, ot, bt, id, cc, tc, tm, mf, tx, ct, tn, dm, dv, kf, fx, dq, nm, ox, px, rx, ui, sy] 6138

6 (trypanosoma adj brucei adj brucei).mp. [mp=ab, hw, ti, ot, bt, id, cc, tc, tm, mf, tx, ct, tn, dm, dv, kf, fx, dq, nm, ox, px, rx, ui, sy] 12705

7 (trypanosoma adj evans*).mp. [mp=ab, hw, ti, ot, bt, id, cc, tc, tm, mf, tx, ct, tn, dm, dv, kf, fx, dq, nm, ox, px, rx, ui, sy] 7762

8 1 or 2 or 3 or 4 or 5 or 6 or 7 61849

9 (knowledge attitude* and practice*).mp. [mp=ab, hw, ti, ot, bt, id, cc, tc, tm, mf, tx, ct, tn, dm, dv, kf, fx, dq, nm, ox, px, rx, ui, sy] 205939

10 drug resistance.mp. [mp=ab, hw, ti, ot, bt, id, cc, tc, tm, mf, tx, ct, tn, dm, dv, kf, fx, dq, nm, ox, px, rx, ui, sy] 890062

11 trypanocide resistance.mp. [mp=ab, hw, ti, ot, bt, id, cc, tc, tm, mf, tx, ct, tn, dm, dv, kf, fx, dq, nm, ox, px, rx, ui, sy] 46

12 resistant trypanocides.mp. [mp=ab, hw, ti, ot, bt, id, cc, tc, tm, mf, tx, ct, tn, dm, dv, kf, fx, dq, nm, ox, px, rx, ui, sy] 1

13 diminazene aceturate.mp. [mp=ab, hw, ti, ot, bt, id, cc, tc, tm, mf, tx, ct, tn, dm, dv, kf, fx, dq, nm, ox, px, rx, ui, sy] 4165
14 (homidium bromide or homidium chloride).mp. [mp=ab, hw, ti, ot, bt, id, cc, tc, tm, mf, tx, ct, tn, dm, dv, kf, fx, dq, nm, ox, px, rx, ui, sy] 1044
15 isometamidium chloride.mp. [mp=ab, hw, ti, ot, bt, id, cc, tc, tm, mf, tx, ct, tn, dm, dv, kf, fx, dq, nm, ox, px, rx, ui, sy] 1409
16 (melarsomine or quinapyramine).mp. [mp=ab, hw, ti, ot, bt, id, cc, tc, tm, mf, tx, ct, sh, tn, dm, dv, kf, fx, dq, nm, ox, px, rx, an, ui, ds, on, sy] 1471
17 10 or 11 or 12 or 13 or 14 or 15 or 16 895932
18 8 and 9 and 17 16
URL: https://www.ezproxy.is.ed.ac.uk/login?url=http://ovidsp.ovid.com/ovidweb.cgi?T=JS&NEWS=N&PAGE=main&SHAREDSEARCHID=6Ofa23UUAuNnmVTJzqDLdc9fXLaliRbMvCjedp7DD9mb2bRyvqvBhIjQH7K1LIoFv

Web of Science

((ALL=(African trypanosomiasis or bovine trypanosomiasis or animal African trypanosomiasis or trypanosoma brucei or trypanosoma brucei brucei or trypanosoma evansi or trypanosoma vivax or trypanosoma congolense)) AND ALL=(knowledge attitude and practices)) AND ALL=(drug resistance or trypanocide resistance or diminazene aceturate or resistant trypanosomes or homidium achloride or homidium bromide or melarsomine or quinupramine or isometamidium chloride)

URL: https://www.webofscience.com/wos/woscc/summary/11c7229b-ff7f-4b7b-9be1-b53794245a19-3ba46071/relevance/1

Summary of search output from databases

AMED (Allied and Complementary Medicine) <1985 to May 2022>	0
CAB Abstracts <1973 to 2022 Week 21>	5
APA PsycInfo <1806 to May Week 4 2022>	0
Books@Ovid <May 31, 2022>	0
Journals@Ovid Full Text <May 31, 2022>	2
Your Journals@Ovid	2
APA PsycArticles Full Text	0
CAB Abstracts <1910 to 1989>	0
Embase Classic+Embase <1947 to 2022 May 31>	1
Global Health <1910 to 2022 Week 21>	4
Ovid MEDLINE(R) and Epub Ahead of Print, In-Process, In-Data-Review & Other Non-Indexed Citations, Daily and Versions <1946 to May 31, 2022>	2
Web of Science	01
Total	17
